# A predictive coding approach to psychedelic virtual-induced hallucinations and creative cognition in aging

**DOI:** 10.3389/fnhum.2023.1219052

**Published:** 2023-07-07

**Authors:** Giulia Magni, Cosimo Tuena, Giuseppe Riva

**Affiliations:** ^1^Applied Technology for Neuro-Psychology Lab, IRCCS Istituto Auxologico Italiano, Milan, Italy; ^2^Humane Technology Lab, Università Cattolica del Sacro Cuore, Milan, Italy

**Keywords:** psychedelics, hallucinations, virtual reality, cognitive flexibility, predictive coding, Bayesian brain approach, aging, mild cognitive impairment

## Abstract

Recent research has investigated the potential of psychedelic substances in treating various neurological and psychiatric disorders. In particular, there has been a growth in studies concerning the intersection of psychedelics, Virtual Reality (VR), and Cognitive Flexibility (CF). Indeed, the use of immersive technology allows the simulation of the perceptual and cognitive effects of psychedelic substances without the potential risks associated with them. CF is strongly associated with creative cognition, a complex cognitive mechanism involved in creative thinking and associated with the prefrontal cortex and the neural networks supporting executive functions, memory, attention, and spontaneous modes of thought. The Bayesian brain approach, which is rooted in predictive coding, has emerged as a promising framework for understanding the effects of psychedelic hallucinations on cognitive functioning. Psychedelic substances may enhance creativity by inducing a state of CF, allowing for a wider range of associations and possibilities to be explored and increasing openness to experience. A decline in cognitive abilities, including creative processing and divergent thinking, is observed during the aging process. In particular, studies on Mild Cognitive Impairment (MCI) show poorer performance in executive functions, including CF. The present paper suggests that psychedelic hallucinations induced by VR may help optimize the balance between top-down expectations and bottom-up sensory information. Therefore, enhanced CF and creativity may be crucial during the aging process for maintaining cognitive functions and preventing pathological conditions.

## Introduction

In recent years, there has been an upsurge in research related to psychedelics. Clinical studies have investigated the therapeutic potential of substances such as psilocybin or Lysergic Acid Diethylamide (LSD), and their role in psychiatric, neurological, and psychological disorders (Vann Jones and O’Kelly, 2020; Doss et al., 2021). Moreover, their potential for cognitive enhancement and their ability to foster neuroplasticity and reduce neuroinflammation has aroused interest (de Vos et al., 2021). Psychedelic substances lead the user to experience altered states of consciousness, which are qualitative alterations in the mental functioning pattern, similar to those experienced when dreaming or engaging in meditative activities (Suzuki et al., 2017). The drug-induced state reminds of Maslow’s (1964) definition of peak experiences, which are characterized by spatial and temporal disorientation, ego transcendence, and the emphasis on the goodness in the world. Therefore, psychedelic experiences may be referred to as transformative experiences and may correlate to enhanced creativity and profound change in the individual (Gaggioli, 2016; Brivio et al., 2020).

However, the use of psychedelic substances in scientific research is hampered by ethical and legal concerns. These drugs are illegal in most countries and obtaining ethical approval for scientific research involving their usage poses difficulties. Moreover, it is easy to confuse the systemic physiological effects of psychedelic drugs with their cognitive effects (Greco et al., 2021). Therefore, technologies – first and foremost Virtual Reality (VR)–have been investigated for the simulation of altered perceptive and affective states (Aday et al., 2020a). This work will outline the potential link between predictive coding mechanisms, virtual-induced hallucinations, and aging.

## Creativity and cognitive flexibility

Most definitions of creativity focus on the novelty, originality, value, and appropriateness of the creative output (Glăveanu, 2021). Creative cognition refers to the cognitive processes involved in creative thinking, encompassing divergent and convergent thinking (Ward, 2007). A key characteristic of creative thinking is cognitive flexibility (CF), which is characterized by a low level of perseveration (i.e., the inability to switch between thinking modes; Dietrich, 2004). CF increases following diversifying and awe-inducing experiences, participating in courses, and taking psychedelic substances (Ritter et al., 2012; Baggott, 2015; Ritter and Mostert, 2017; Chirico et al., 2018). Indeed, CF is the ability to adapt to various cognitive functions and respond to changing environmental demands, and its measurement often consists of evaluating the perseveration of previous rules in different tasks (Uddin, 2021).

Neuroscientific research suggests a multifaced nature of the creative process, which relies on high-level cognitive operations rather than specific brain regions or hemispheres (Dietrich and Kanso, 2010). The neural system supporting creativity involves a diverse network of brain regions across both hemispheres and is facilitated by open-ended challenges in various domains (Boccia et al., 2015). Indeed, interhemispheric interaction is essential for creative processes, which engage functionally specialized brain areas rather than specific regions (Dietrich and Kanso, 2010). The Geneplore model provides a framework for creative cognition, emphasizing generative processes in constructing visual ideas and exploratory processes in examining and interpreting those ideas (Finke et al., 1992; Smith et al., 1995). Therefore, different domains of creativity require functional specialization, such as verbal creativity being predominantly domain-specific but also influenced by the visual domain (Boccia et al., 2015), visual creativity being contingent on the specific domain and task (Palmiero et al., 2010), and mental imagery relying on perceptual information accessed from memory (Palmiero et al., 2011). Musical and artistic creativity are associated with altered states of mind linked to executive functions (EFs, i.e., fluency and flexibility; Dietrich, 2004).

The prefrontal cortex (PFC), responsible for voluntary, goal-directed behavior and adaptive responses to context, plays a fundamental role in creative thinking (Miller and Cohen, 2001; Levy and Volle, 2009). Impairments in the PFC are generally associated with reduced creative abilities, although some cases with brain damage in this region experience enhanced aspects of creativity (Fink and Benedek, 2019). The dorsolateral PFC (DLPFC) is consistently involved in problem-solving, monitoring, and focused attention across creative domains (Dietrich, 2004). Specifically, verbal creativity activates the inferior frontal gyrus, and both musical and verbal processes engage the right posterior cerebellum, default network, temporal, parietal, and occipital areas (Ellamil et al., 2012; Boccia et al., 2015). Memory processes also contribute to creativity, involving the medial temporal lobe (Boccia et al., 2015). Visuospatial creativity primarily relies on the bilateral thalamus and premotor cortices, without engaging temporal, parietal, and occipital regions (Boccia et al., 2015).

Thus, brain areas activated during the creative process are the same as those involved in normal cognitive processes, suggesting integration into normal cognition, and a stronger functional brain connectivity between neural circuits related to creativity has been observed in creative individuals (Beaty et al., 2018; Benedek and Fink, 2019). Creative cognition is therefore associated with secondary aspects such as EFs, memory, and focused attention (Beaty et al., 2019). Focused attention enhances awareness of the environment and internal physiological state, with implications for memory, attention, and executive control (Campbell, 1960; Mednick, 1962; Benedek and Fink, 2019; Kitson et al., 2019, 2022). Controlled and spontaneous cognitive processes interact in creative cognition, as evidenced by the connectivity between default brain networks and executive control networks (Beaty et al., 2016). However, the presence of active but less-focused attention, as opposed to tightly focused attention, has also been observed as beneficial for the heightened state of creative awareness. For instance, research has shown that an active attentional state coupled with loose probabilistic and temporal expectancies on forthcoming conscious events can facilitate the awareness of visual events that would otherwise go unnoticed (Lasaponara et al., 2015). Similar outcomes have been successfully observed in the spatial domain (Lasaponara et al., 2020). The interplay between conscious and unconscious processing adapts dynamically based on the probabilistic properties of the sensory environment, and attentional conditions that facilitate unexpected discoveries align with the effects of psychedelic substances.

## Bayesian interpretation of psychedelic hallucinations, creativity, and virtual reality

Virtual Reality-simulated hallucinations have been explained through different theoretical approaches. According to some authors (Brivio et al., 2020; Greco et al., 2021; Rastelli et al., 2022), hallucinations are effective not only because they alter the external world’s perception, but also because they lead to enhanced brain entropy. Indeed, the Entropic Brain Hypothesis states that psychedelic experiences increase entropy and functional connectivity pattern. Aday et al. (2020b) traces a parallel between psychedelics and VR, stating that they both work by enhancing flexible sensory perception and altering visual processing.

An additional theoretical approach is the Bayesian Brain Approach, which is a framework rooted in predictive coding that aims at predicting and understanding human perception and cognition (Clark, 2013; Suzuki et al., 2017; Leach, 2022). Hutchinson and Barrett (2019) argue that sensory information is combined with prior beliefs and expectations to generate probabilistic predictions, according to a top-down process that starts in the brain. Specifically, perception and inference are based on the representation of both the likelihood and prior probability of different hypotheses (Friston et al., 2006).

Hence, the Bayesian model has emerged as a promising approach to understanding the effects of psychedelic hallucinations on cognitive functioning. Corlett et al. (2009) suggest that psychedelic substances may influence the Bayesian process by reducing the reliability of incoming bottom-up signals while preserving top-down signals that can add structure to the bottom-up signal. In turn, this disruption may facilitate the generation of novel and unconventional ideas, leading to increased creativity (Harman et al., 1966; Barron and Harrington, 1981). In other words, according to the Bayesian model, creativity may arise from the ability to make predictions that are both precise and flexible, such that unexpected or novel sensory input can be integrated in a meaningful way (Hutchinson and Barrett, 2019). Psychedelics may enhance creativity by inducing a state of CF in which the usual constraints on perception and thought are loosened, allowing for a wider range of associations to be explored (Carhart-Harris and Friston, 2019). This idea is supported by studies showing that psychedelic use is associated with increased openness to experience, a trait that has been linked to creative thinking (MacLean et al., 2011; Nour et al., 2016).

A way to alter the predictive coding mechanisms of the brain is through the use of VR tasks. Indeed, VR is capable to alter top-down or bottom-up information and modify the brain’s inference processes (Riva et al., 2019).

According to neuroscience, in order to effectively regulate and control the body, the brain creates an embodied simulation of the body in the world that is used to represent and predict actions, concepts, and emotions (Riva et al., 2017, 2021). VR operates in a similar fashion: the VR experience attempts to predict the consequences of an individual’s actions, thoughts, and even emotions, providing them with the same scene they would experience in the real world. To achieve this, the VR system, like the brain, maintains a model (simulation) of the body and the space around it. Different studies show that VR and innovative technologies can alter Bayesian inference and embodied mechanisms in different populations (Riva and Dakanalis, 2018; Riva et al., 2021; Tuena et al., 2022; Di Lernia et al., 2023). Indeed, innovative technologies combined with VR can be seen as the future of the so-called regenerative virtual therapy medicine (Riva et al., 2017).

## Deep Dream and simulated hallucinations

Big data are defined as information assets characterized by high volume, high velocity, and high variety. They necessitate efficient and innovative methods of information processing to improve insight, decision-making, and process automation. In itself, big data is abstract, but its various applications lead to useful and promising purposes, such as cost and time optimization (Dahlstedt, 2019).

**FIGURE 1:**
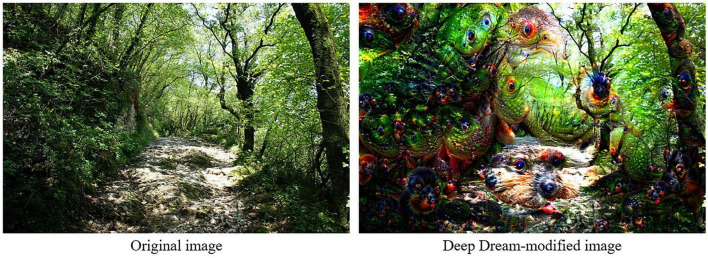
Example of visual stimuli presented in VR. Panoramic video frame **(left)** and its counterpart modified with Deep Dream with Inception Depth set as “deep” and Neural Network Layer as 15 **(right)**.

An example of big data is Deep Dream (DD), an open-source software released by Google in 2015 consisting of a deep learning technological system that generates images. Frequently, the outcome consists of pictures that are characterized by being novel, unusual, and with hallucinatory-like qualities (Boden, 2017). Indeed, a similarity was observed between these images and psychedelic hallucinations produced by LSD or psilocybin intake, where different shapes and elements morph and blend into each other (Chatonsky, 2016). The brain actively participates in the perception of the external world by making predictions (Riva et al., 2018). Similarly, DD-generated hallucinations are the result of a process of interpretation and prediction of the external world on the basis of a specific dataset (Leach, 2022). DD is Google’s approach to the visualization of a specific Deep Neural Network architecture, called Convolutional Neural Networks (CNN). Trained DCNNs have high complexity and include various parameters and nodes, and are used as a method to increase the pattern recognition tasks’ complexity, through the combination of multiple layers containing several functional units (Suzuki et al., 2017; Weißkirchen et al., 2017). An example of the DD-hallucinated images can be observed in Figure 1, which was obtained by using the non-modified image as the input on the Deep Dream website.^1^ DD works through multiple iterations of a single process: an image is given to the deep learning network, in which the system discovers patterns and emphasizes said features through image modification (Boden, 2017).

Due to DD’s ability to reproduce visual hallucinations, it has been employed by different studies in order to investigate its potential for cognitive aspects such as creativity and relaxation (e.g., Kitson et al., 2019; Brivio et al., 2020; Rastelli et al., 2022).

On the one hand, the DD-modified images have been associated with hallucinations experienced by schizophrenic patients (Ando et al., 2011). Therefore, researchers have investigated its role in the destigmatization of the disease, which resulted in an enhanced understanding of and empathy toward psychotic disorders (Ando et al., 2011; Riches et al., 2018).

On the other hand, various studies have employed these hallucination-like images to investigate the role of altered states of consciousness frequently induced by psychedelic substances (Suzuki et al., 2017, 2018; Kitson et al., 2019, 2022; Brivio et al., 2020; Rastelli et al., 2022). In this regard, Suzuki et al. (2018) ideated the Hallucination Machine, aiming at achieving a better understanding of human conscious phenomenology. Through the combination of VR and machine learning, they developed a technique that differentiates the effect of visual hallucinations from the secondary systemic physiological effects of the substances. Specifically, the Hallucination Machine applies the DD algorithm to panoramic 360° videos of natural scenes, which are then presented to the user through a head-mounted display. The subjective experience was found qualitatively similar to pharmacologically-induced psychedelic states, specifically those deriving from psilocybin administration. However, temporal distortions were not noted (Suzuki et al., 2017).

The Hallucination Machine technique was employed in several studies. Rastelli et al. (2022) built on the hypothesis that psychedelic experiences play a significant role in modulating CF. The participants were administered the Alternative Uses Task (AUT) and a mouse-tracking version of the Stroop task, as well as the Altered States of Consciousness questionnaire following the experience. The results confirmed Suzuki’s hypothesis that the virtual experience was qualitatively similar to that of psilocybin. In spite of non-significant improvement in the Stroop task performance, which might be explained by the fact that DD modulated different cognitive aspects that might have interfered, AUT results confirmed increased CF.

Similarly, Brivio et al. (2020) investigated the Hallucination Machine’s use to stimulate creativity. The study demonstrated an improvement as a result of the 360° video exposition. However, no significant effects resulted from the hallucinatory distortion, possibly due to the undermining of the experience’s effectiveness in increasing brain entropy from a multisensory conflict between outside and inside inputs.

The Lucid Loop program integrated the Hallucination Machine technique with biofeedback and neurofeedback methods that include the measurements of brain waves, heart rate, and respiration, to simulate a lucid dreaming experience and enhance awareness. Indeed, biofeedback techniques are frequently used to practice awareness of physiological states and to train one’s own physiological response patterns (Kitson et al., 2019). Results showed Lucid Loop to be a significant experience to support focused attention and foster strong emotional responses (Kitson et al., 2022).

## Linking aging, predictive coding, and virtual-induced hallucinations

The normal evolution of aging involves a decline in most cognitive functions, including creative processing (Price and Tinker, 2014; Buitenweg et al., 2019). The frontal lobes, which are critically involved in divergent thinking, exhibit the highest rate of atrophy in non-pathological aging. Indeed, it implies a reduction in white matter connectivity, a key aspect of normal frontal lobe functioning (Guttmann et al., 1998; Damasio and Anderson, 2003). These age-related anatomic changes are associated with a decline in frontally-mediated tasks (Leon et al., 2014). Additionally, creative cognition contributes to many activities that naturally decline with age, such as problem-solving, flexible thinking, and memory retrieval (de Souza et al., 2010; Price and Tinker, 2014), making it an essential ability for other cognitive activities and for everyday functioning (Baldassini et al., 2017; Buitenweg et al., 2019).

Mild Cognitive Impairment (MCI) is often considered a transitional stage between normal aging and dementia. With the progression of MCI, executive dysfunction becomes more prominent (Kirova et al., 2015). Studies have shown that individuals in the early stage of MCI exhibit poorer performance in EF tasks compared to healthy individuals (Seo et al., 2016). EFs encompass higher cognitive functions such as inhibitory control, CF, and working memory, which are mainly supported by the PFC and are among the first executive components affected in the prodromal stage of Alzheimer’s disease (AD) (Traykov et al., 2007). In this regard, EF impairment has been observed in MCI patients and is associated with a higher risk of conversion to dementia (Reinvang et al., 2012; Jung et al., 2020). Additionally, structural brain changes in regions involved in EFs (i.e., PFC, entorhinal cortex, hippocampus) have been observed in individuals with MCI, supporting the association between CF impairments and neuroanatomical alterations (Corbo and Casagrande, 2022). The neurophysiological changes associated with aging and MCI involve a shift toward increased reliance on internal predictions or priors, resulting in an altered balance between predictions and sensory evidence (Kocagoncu et al., 2021). These findings highlight the significance of CF and EF impairments in aging processes and MCI, indicating their potential as early markers and predictors of cognitive decline and progression to dementia (Ready et al., 2003; Rapp, 2005).

According to the predictive coding model, hallucinations may be related to a discrepancy between top-down predictions and bottom-up sensory input, leading to perceptual distortions. This process can help foster CF, which role is fundamental in the aging process since it allows the individual to update and refine prior expectations in response to changing environments (Harman et al., 1966; Barron and Harrington, 1981; Friston et al., 2006). The Bayesian model states that the aging brain selectively attenuates prediction errors in a hierarchy-selective manner, which may lead to reduced sensitivity to new or unexpected information (Gilbert and Moran, 2016; Hsu et al., 2021). Specifically, dysfunction in the predictive coding system, such as the attenuation of prediction errors in higher-order areas, has been implicated in cognitive decline and dementia. For instance, disruption of predictive coding mechanisms can be observed in dementia, which may result in aberrant sensory processing, impaired perception, and distorted beliefs (Kocagoncu et al., 2021). Frontotemporal dementia has been associated with a state of allostatic-interoceptive overload, in which the balance between prior expectations and sensory input is disturbed, leading to distorted and maladaptive predictions (Migeot et al., 2022). These findings highlight the importance of maintaining a healthy balance between prior expectations and bottom-up sensory information in healthy aging and of rehabilitating any imbalances in pathological aging.

**FIGURE 2:**
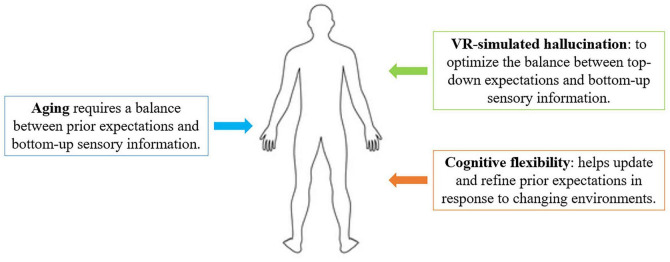
Relationship between VR-simulated hallucinations and aging.

The simulation of psychedelic hallucinations may help promote CF through the optimization of the balance between top-down expectations and bottom-up sensory information, as illustrated in Figure 2. Different studies have demonstrated the potential for hallucinations to foster creativity and CF, as well as their clinical therapeutic potential (de Vos et al., 2021). Since CF and creative thinking are both linked to the PFC, they might influence different cognitive functions, such as EFs and memory processes (Miller and Cohen, 2001; Levy and Volle, 2009). Supporting and cultivating CF and creative thinking during the aging process could therefore reveal crucial to maintain cognitive functions and preventing pathological conditions. Additionally, VR-simulated hallucinations could also be a valid and useful tool for mild dementia and in the initial stages following possible conversion from MCI to dementia.

## Conclusion

The Bayesian brain approach to hallucinations provides a framework to understand psychedelic substances’ effects on cognition. By disrupting the usual constraints on perception and thought, hallucinatory phenomena might enhance CF and creativity. The predictive coding model suggests that psychedelic hallucinations may help contrast the decline of these abilities both in healthy and pathological aging processes, by optimizing the balance between top-down expectations and bottom-up sensory information. In particular, it could be interesting to explore their role in the transition between healthy aging and MCI and in MCI conversion to dementia.

New technologies such as VR could help overcome the ethical and legal limits posed by the use of psychedelics and further examine their effects on cognitive functions. Indeed, simulated hallucinations have shown successful results in improving CF, but the results are rarely generalizable due to small sample sizes and self-report measures of creativity. Future studies might focus on a target group of older adults, in order to explore the psychedelic potential for aging. Furthermore, the implementation of neuro- and biofeedback systems could be considered to modulate the hallucinatory phenomenon through physiological reactions and signals, as well as the personalization of the input to the machine learning framework (e.g., participants could adjust the hallucinations’ level of abstraction and category type to have a more realistic experience). Specifically, by choosing different inputs one could have better control of the type of hallucination obtained, in order to reach certain results and manage the experience.

## Data availability statement

The original contributions presented in this study are included in this article/supplementary material, further inquiries can be directed to the corresponding author.

## Author contributions

GM and CT: conceptualization, writing—first draft, and editing. GR: supervision. All authors contributed to the conception and design of the study, manuscript revision, and read and approved the submitted version.

## Footnotes

1
https://deepdreamgenerator.com

## References

[B1] Aday J. S. Davoli C. C. Bloesch E. K. (2020a). Psychedelics and virtual reality: Parallels and applications. *Ther. Adv. Psychopharmacol.* 10:204512532094835. 10.1177/2045125320948356 32922734 PMC7446267

[B2] Aday J. S. Mitzkovitz C. M. Bloesch E. K. Davoli C. C. Davis A. K. (2020b). Long-term effects of psychedelic drugs: A systematic review. *Neurosci. Biobehav. Rev*. 113, 179–189. 32194129 10.1016/j.neubiorev.2020.03.017

[B3] Ando S. Clement S. Barley E. A. Thornicroft G. (2011). The simulation of hallucinations to reduce the stigma of schizophrenia: A systematic review. *Schizophr. Res.* 133 8–16. 10.1016/j.schres.2011.09.011 22005017

[B4] Baggott M. J. (2015). Psychedelics and creativity: A review of the quantitative literature. *PeerJ PrePrints* 3:e1202v1. 10.7287/peerj.preprints.1202v1

[B5] Baldassini D. Colombo V. Mottura S. Sacco M. Colautti L. Antonietti A. (2017). “Design of a ICT-based training system to improve creative thinking in brain-damaged patients,” in *Proceedings of the international conference on virtual rehabilitation (ICVR)*, (Piscataway, NJ: IEEE), 1–2.

[B6] Barron F. Harrington D. M. (1981). Creativity, intelligence, and personality. *Annu. Rev. Psychol.* 32 439–476.

[B7] Beaty R. E. Benedek M. Silvia P. J. Schacter D. L. (2016). Creative cognition and brain network dynamics. *Trends Cogn. Sci.* 20 87–95. 10.1016/j.tics.2015.10.004 26553223 PMC4724474

[B8] Beaty R. E. Kenett Y. N. Christensen A. P. Rosenberg M. D. Benedek M. Chen Q. (2018). Robust prediction of individual creative ability from brain functional connectivity. *Proc. Natl. Acad. Sci*. 115, 1087–1092. 29339474 10.1073/pnas.1713532115 PMC5798342

[B9] Beaty R. E. Seli P. Schacter D. L. (2019). Network neuroscience of creative cognition: Mapping cognitive mechanisms and individual differences in the creative brain. *Curr. Opin. Behav. Sci.* 27 22–30. 10.1016/j.cobeha.2018.08.013 30906824 PMC6428436

[B10] Benedek M. Fink A. (2019). Toward a neurocognitive framework of creative cognition: The role of memory, attention, and cognitive control. *Curr. Opin. Behav. Sci.* 27 116–122. 10.1016/j.cobeha.2018.11.002

[B11] Boccia M. Piccardi L. Palermo L. Nori R. Palmiero M. (2015). Where do bright ideas occur in our brain? Meta-analytic evidence from neuroimaging studies of domain-specific creativity. *Front. Psychol.* 6:1195. 10.3389/fpsyg.2015.01195 26322002 PMC4531218

[B12] Boden M. A. (2017). Is deep dreaming the new collage? *Conn. Sci.* 29 268–275. 10.1080/09540091.2017.1345855

[B13] Brivio E. Di Lernia D. Chirico A. Caroli F. Luisi A. Palomba G. (2020). Deep-dream 360^°^ Virtual Reality videos for stimulating creativity: A pilot study. *Annu. Rev. Cyberther. Telemed.* 18 251–255.

[B14] Buitenweg J. I. V. Van De Ven R. M. Ridderinkhof K. R. Murre J. M. J. (2019). Does cognitive flexibility training enhance subjective mental functioning in healthy older adults? *Neuropsychol. Dev. Cogn. B Aging Neuropsychol. Cogn.* 26 688–710. 10.1080/13825585.2018.1519106 30221578

[B15] Campbell D. T. (1960). Blind variation and selective retentions in creative thought as in other knowledge processes. *Psychol. Rev*. 67:380. 10.1037/h0040373 13690223

[B16] Carhart-Harris R. L. Friston K. J. (2019). REBUS and the anarchic brain: Toward a unified model of the brain action of psychedelics. *Pharmacol. Rev.* 71 316–344. 10.1124/pr.118.017160 31221820 PMC6588209

[B17] Chatonsky G. (2016). Deep dream (The network’s dream). *SubStance* 45 61–77.

[B18] Chirico A. Glaveanu V. P. Cipresso P. Riva G. Gaggioli A. (2018). Awe enhances creative thinking: An experimental study. *Creat. Res. J.* 30 123–131. 10.1080/10400419.2018.1446491

[B19] Clark A. (2013). Whatever next? Predictive brains, situated agents, and the future of cognitive science. *Behav. Brain Sci.* 36 181–204. 23663408 10.1017/S0140525X12000477

[B20] Corbo I. Casagrande M. (2022). Higher-level executive functions in healthy elderly and mild cognitive impairment: A systematic review. *J. Clin. Med.* 11:1204. 10.3390/jcm11051204 35268294 PMC8911402

[B21] Corlett P. R. Frith C. D. Fletcher P. C. (2009). From drugs to deprivation: A Bayesian framework for understanding models of psychosis. *Psychopharmacology* 206 515–530. 10.1007/s00213-009-1561-0 19475401 PMC2755113

[B22] Dahlstedt P. (2019). Big data and creativity. *Eur. Rev.* 27 411–439. 10.1017/S1062798719000073

[B23] Damasio A. R. Anderson S. W. (2003). “The frontal lobes,” in *Clinical neuropsychology*, eds Heilman K. M. Valenstein E. (New York, NY: Oxford University Press), 404–446. 10.1111/j.1460-9568.2009.07030.x

[B24] de Souza L. C. Volle E. Bertoux M. Czernecki V. Funkiewiez A. Allali G. (2010). Poor creativity in frontotemporal dementia: A window into the neural bases of the creative mind. *Neuropsychologia* 48 3733–3742. 10.1016/j.neuropsychologia.2010.09.010 20868703

[B25] de Vos C. M. H. Mason N. L. Kuypers K. P. C. (2021). Psychedelics and neuroplasticity: A systematic review unraveling the biological underpinnings of psychedelics. *Front. Psychiatry* 12:724606. 10.3389/fpsyt.2021.724606 34566723 PMC8461007

[B26] Di Lernia D. Serino S. Tuena C. Cacciatore C. Polli N. Riva G. (2023). Mental health meets computational neuroscience: A predictive Bayesian account of the relationship between interoception and multisensory bodily illusions in anorexia nervosa. *Int. J. Clin. Health Psychol.* 23:100383. 10.1016/j.ijchp.2023.100383 36937547 PMC10017360

[B27] Dietrich A. (2004). The cognitive neuroscience of creativity. *Psychon. Bull. Rev.* 11 1011–1026. 10.3758/BF03196731 15875970

[B28] Dietrich A. Kanso R. (2010). A review of EEG, ERP, and neuroimaging studies of creativity and insight. *Psychol. Bull.* 136 822–848. 10.1037/a0019749 20804237

[B29] Doss M. K. Považan M. Rosenberg M. D. Sepeda N. D. Davis A. K. Finan P. H. (2021). Psilocybin therapy increases cognitive and neural flexibility in patients with major depressive disorder. *Transl. Psychiatry* 11:574. 10.1038/s41398-021-01706-y 34750350 PMC8575795

[B30] Ellamil M. Dobson C. Beeman M. Christoff K. (2012). Evaluative and generative modes of thought during the creative process. *Neuroimage* 59, 1783–1794. 21854855 10.1016/j.neuroimage.2011.08.008

[B31] Fink A. Benedek M. (2019). The neuroscience of creativity. *Neuroforum* 25 231–240. 10.1515/nf-2019-0006

[B32] Finke R. A. Ward T. B. Smith S. M. (1992). *Creative cognition: Theory, research, and applications.* Cambridge, MA: MIT Press.

[B33] Friston K. Kilner J. Harrison L. (2006). A free energy principle for the brain. *J. Physiol. Paris* 100 70–87. 17097864 10.1016/j.jphysparis.2006.10.001

[B34] Gaggioli A. (2016). “Transformative experience design,” in *Human computer confluence. Transforming human experience through symbiotic technologies*, eds Gaggioli A. Ferscha A. Riva G. Dunne S. Viaud-Delmon I. (Warsaw: De Gruyter Open Poland), 97–122.

[B35] Gilbert J. R. Moran R. J. (2016). Inputs to prefrontal cortex support visual recognition in the aging brain. *Sci. Rep.* 6:31943. 10.1038/srep31943 27550752 PMC4994026

[B36] Glăveanu V. P. (2021). *Creativity: A very short introduction.* Oxford: Oxford University Press.

[B37] Greco A. Gallitto G. D’Alessandro M. Rastelli C. (2021). Increased entropic brain dynamics during deepdream-induced altered perceptual phenomenology. *Entropy* 23:839. 10.3390/e23070839 34208923 PMC8306862

[B38] Guttmann C. R. G. Jolesz F. A. Kikinis R. Killiany R. J. Moss M. B. Sandor T. (1998). White matter changes with normal aging. *Neurology* 50 972–978. 10.1212/WNL.50.4.972 9566381

[B39] Harman W. W. McKim R. H. Mogar R. E. Fadiman J. Stolaroff M. J. (1966). Psychedelic agents in creative problem-solving: A pilot study. *Psychol. Rep.* 19 211–227. 10.2466/pr0.1966.19.1.211 5942087

[B40] Hsu Y. F. Waszak F. Strömmer J. Hämäläinen J. A. (2021). Human brain ages with hierarchy-selective attenuation of prediction errors. *Cereb. Cortex* 31 2156–2168. 10.1093/cercor/bhaa352 33258914 PMC7945026

[B41] Hutchinson J. B. Barrett L. F. (2019). The power of predictions: An emerging paradigm for psychological research. *Curr. Dir. Psychol. Sci.* 28 280–291. 31749520 10.1177/0963721419831992 PMC6867616

[B42] Jung Y. H. Park S. Jang H. Cho S. H. Kim S. Kim J. P. (2020). Frontal-executive dysfunction affects dementia conversion in patients with amnestic mild cognitive impairment. *Sci. Rep.* 10:772. 10.1038/s41598-020-57525-6 31964931 PMC6972894

[B43] Kirova A.-M. Bays R. B. Lagalwar S. (2015). Working memory and executive function decline across normal aging, mild cognitive impairment, and Alzheimer’s disease. *BioMed Res. Int.* 2015 1–9. 10.1155/2015/748212 26550575 PMC4624908

[B44] Kitson A. DiPaola S. Riecke B. E. (2019). “Lucid loop: A virtual deep learning biofeedback system for lucid dreaming practice,” in *Proceedings of the extended abstracts of the 2019 CHI conference on human factors in computing systems*, (New York, NY: Association for Computing Machinery), 1–6. 10.1145/3290607.3312952

[B45] Kitson A. Muntean R. DiPaola S. Riecke B. E. (2022). “Lucid loop: Exploring the parallels between immersive experiences and lucid dreaming,” in *Proceedings of the designing interactive systems conference*, (New York, NY: Association for Computing Machinery, Inc.), 865–880. 10.1145/3532106.3533538

[B46] Kocagoncu E. Klimovich-Gray A. Hughes L. E. Rowe J. B. (2021). Evidence and implications of abnormal predictive coding in dementia. *Brain* 144 3311–3321. 10.1093/brain/awab254 34240109 PMC8677549

[B47] Lasaponara S. Dragone A. Lecce F. Di Russo F. Doricchi F. (2015). The “serendipitous brain”: Low expectancy and timing uncertainty of conscious events improve awareness of unconscious ones (evidence from the Attentional Blink). *Cortex* 71 15–33. 10.1016/j.cortex.2015.05.029 26142182

[B48] Lasaponara S. Pinto M. Pellegrino M. Caratelli L. Rossi-Arnaud C. Cestari V. (2020). Spatial uncertainty improves the distribution of visual attention and the availability of sensory information for conscious report. *Exp. Brain Res.* 238 2031–2040. 10.1007/s00221-020-05862-3 32617884

[B49] Leach N. (2022). Architectural hallucinations: What can ai tell us about the mind of an architect? *Archit. Design* 92 66–71. 10.1002/ad.2815

[B50] Leon S. A. Altmann L. J. Abrams L. Rothi L. J. G. Heilman K. M. (2014). Divergent task performance in older adults: Declarative memory or creative potential? *Creat. Res. J.* 26 21–29. 10.1080/10400419.2014.873657 28446859 PMC5403144

[B51] Levy R. Volle E. (2009). The prefrontal cortex: Composer and conductor of voluntary behaviors. *Rev. Neurol.* 165 159–177. 20222196

[B52] MacLean K. A. Johnson M. W. Griffiths R. R. (2011). Mystical experiences occasioned by the hallucinogen psilocybin lead to increases in the personality domain of openness. *J. Psychopharmacol.* 25 1453–1461. 10.1177/0269881111420188 21956378 PMC3537171

[B53] Maslow A. (1964). *Religions, values and peak experiences.* Columbus, OH: Ohio State University Press.

[B54] Mednick S. (1962). The associative basis of the creative process. *Psychol. Rev*. 69:220. 10.1037/h0048850 14472013

[B55] Migeot J. A. Duran-Aniotz C. A. Signorelli C. M. Piguet O. Ibáñez A. (2022). A predictive coding framework of allostatic–interoceptive overload in frontotemporal dementia. *Trends Neurosci.* 45 838–853. 10.1016/j.tins.2022.08.005 36057473 PMC11286203

[B56] Miller E. K. Cohen J. D. (2001). An integrative theory of prefrontal cortex function. *Annu. Rev. Neurosci.* 24 167–202. 11283309 10.1146/annurev.neuro.24.1.167

[B57] Nour M. M. Evans L. Nutt D. Carhart-Harris R. L. (2016). Ego-dissolution and psychedelics: Validation of the ego-dissolution inventory (EDI). *Front. Hum. Neurosci.* 10:269. 10.3389/fnhum.2016.00269 27378878 PMC4906025

[B58] Palmiero M. Cardi V. Belardinelli M. O. (2011). The role of vividness of visual mental imagery on different dimensions of creativity. *Creat. Res. J.* 23 372–375. 10.1080/10400419.2011.621857

[B59] Palmiero M. Nakatani C. Raver D. Belardinelli M. O. van Leeuwen C. (2010). Abilities within and across visual and verbal domains: How specific is their influence on creativity? *Creat. Res. J.* 22 369–377. 10.1080/10400419.2010.523396

[B60] Price K. A. Tinker A. M. (2014). Creativity in later life. *Maturitas* 78 281–286. 10.1016/j.maturitas.2014.05.025 24974278

[B61] Rapp M. A. (2005). Attention and executive control predict alzheimer disease in late life: Results from the berlin aging study (BASE). *Am. J. Geriatr. Psychiatry* 13 134–141. 10.1176/appi.ajgp.13.2.134 15703322

[B62] Rastelli C. Greco A. Kenett Y. N. Finocchiaro C. De Pisapia N. (2022). Simulated visual hallucinations in virtual reality enhance cognitive flexibility. *Sci. Rep.* 12:4027. 10.1038/s41598-022-08047-w 35256740 PMC8901713

[B63] Ready R. E. Ott B. R. Grace J. Cahn-Weiner D. A. (2003). Apathy and executive dysfunction in mild cognitive impairment and Alzheimer disease. *Am. J. Geriatr. Psychiatry* 11 222–228. 12611752

[B64] Reinvang I. Grambaite R. Espeseth T. (2012). Executive dysfunction in MCI: Subtype or early symptom. *Int. J. Alzheimers Dis.* 2012 1–8. 10.1155/2012/936272 22693679 PMC3369514

[B65] Riches S. Maskey R. Waddingham R. Benjamin J. Dishman P. Tebrook C. (2018). Altered states of consciousness: Evaluation of a voice-hearing simulation during an immersive art exhibition. Early Intervent. *Psychiatry* 12, 947–950. 10.1111/eip.12497 29116669

[B66] Ritter S. M. Mostert N. (2017). Enhancement of creative thinking skills using a cognitive-based creativity training. *J. Cogn. Enhanc.* 1 243–253. 10.1007/s41465-016-0002-3

[B67] Ritter S. M. Damian R. I. Simonton D. K. van Baaren R. B. Strick M. Derks J. (2012). Diversifying experiences enhance cognitive flexibility. *J. Exp. Soc. Psychol.* 48 961–964. 10.1016/j.jesp.2012.02.009

[B68] Riva G. Dakanalis A. (2018). Altered processing and integration of multisensory bodily representations and signals in eating disorders: A possible path toward the understanding of their underlying causes. *Front. Hum. Neurosci.* 12:49. 10.3389/fnhum.2018.00049 29483865 PMC5816057

[B69] Riva G. Serino S. Di Lernia D. Pagnini F. (2021). Regenerative virtual therapy: The use of multisensory technologies and mindful attention for updating the altered representations of the bodily self. *Front. Syst. Neurosci.* 15:749268. 10.3389/fnsys.2021.749268 34803617 PMC8595209

[B70] Riva G. Serino S. Di Lernia D. Pavone E. F. Dakanalis A. (2017). Embodied medicine: Mens sana in corpore virtuale sano. *Front. Hum. Neurosci.* 11:120. 10.3389/fnhum.2017.00120 28360849 PMC5352908

[B71] Riva G. Wiederhold B. K. Mantovani F. (2019). Neuroscience of virtual reality: From virtual exposure to embodied medicine. *Cyberpsychol. Behav. Soc. Netw.* 22 82–96. 30183347 10.1089/cyber.2017.29099.gri PMC6354552

[B72] Riva G. Wiederhold B. K. Chirico A. Di Lernia D. Mantovani F. Gaggioli A. (2018). Brain and virtual reality: What do they have in common and how to exploit their potential. *Annu. Rev. Cyberther. Telemed.* 16 3–7.

[B73] Seo E. H. Kim H. Lee K. H. Choo I. H. (2016). Altered executive function in pre-mild cognitive impairment. *J. Alzheimers Dis.* 54 933–940. 10.3233/JAD-160052 27567814

[B74] Smith S. M. Ward T. B. Finke R. A. (1995). *The creative cognition approach.* Cambridge, MA: The MIT Press.

[B75] Suzuki K. Roseboom W. Schwartzman D. J. Seth A. K. (2017). A deep-dream virtual reality platform for studying altered perceptual phenomenology. *Sci. Rep.* 7:15982. 10.1038/s41598-017-16316-2 29167538 PMC5700081

[B76] Suzuki K. Roseboom W. Schwartzman D. J. Seth A. K. (2018). “Hallucination machine: Simulating altered perceptual phenomenology with a deep-dream virtual reality platform,” in *Proceedings of the ALIFE 2018: The 2018 conference on artificial life*, (Cambridge, MA: MIT Press), 111–112.

[B77] Traykov L. Raoux N. Latour F. Gallo L. Hanon O. Baudic S. (2007). Executive functions deficit in mild cognitive impairment. *Cogn. Behav. Neurol.* 20 219–224. 10.1097/WNN.0b013e31815e6254 18091070

[B78] Tuena C. Riva G. Murru I. Campana L. Goulene K. M. Pedroli E. (2022). Contribution of cognitive and bodily navigation cues to egocentric and allocentric spatial memory in hallucinations due to Parkinson’s disease: A case report. *Front. Behav. Neurosci.* 16:992498. 10.3389/fnbeh.2022.992498 36311858 PMC9606325

[B79] Uddin L. Q. (2021). Cognitive and behavioural flexibility: Neural mechanisms and clinical considerations. *Nat. Rev. Neurosci.* 22 167–179. 10.1038/s41583-021-00428-w 33536614 PMC7856857

[B80] Vann Jones S. A. O’Kelly A. (2020). Psychedelics as a treatment for Alzheimer’s disease dementia. *Front. Synaptic Neurosci.* 12:34. 10.3389/fnsyn.2020.00034 32973482 PMC7472664

[B81] Ward T. B. (2007). Creative cognition as a window on creativity. *Methods* 42 28–37. 10.1016/j.ymeth.2006.12.002 17434413

[B82] Weißkirchen N. Bock R. Wendemuth A. (2017). “Recognition of emotional speech with convolutional neural networks by means of spectral estimates,” in *Proceedings of the 2017 seventh international conference on affective computing and intelligent interaction workshops and demos (ACIIW)*, (Piscataway, NJ: IEEE), 50–55.

